# Transcriptome profiling of gene expression in fall dormant and nondormant alfalfa

**DOI:** 10.1016/j.gdata.2014.08.008

**Published:** 2014-09-06

**Authors:** Senhao Zhang, Chengzhang Wang

**Affiliations:** College of Animal Science and Veterinary Medicine, Henan Agricultural University, Zhengzhou, Henan 450002, China

**Keywords:** Alfalfa, RNA-seq, Differential gene expression, Fall dormancy

## Abstract

Fall dormancy (FD) is an adaptive trait in alfalfa (*Medicago sativa* L.). It appeared in the regrowth process in late summer or early autumn after alfalfa was harvested. FD affects the biomass accumulation and winter survival in high latitude area. However, the molecular mechanism under FD is still not clear at present. Performing RNA-seq of fall dormant and nondormant alfalfa varieties at different time points, we obtained differentially expressed genes between different FD types or time points. These differentially expressed genes may relate to FD in alfalfa. Here, we provide detailed experimental methods and analysis pipeline in our study (Zhang S et al., De novo Characterization of Fall Dormant and Nondormant Alfalfa (*Medicago sativa* L.) Transcriptome and Identification of Candidate Genes Relate to Fall Dormancy, submitted for publication) for reproducible research. Data generated in our work provide a resource to help decipher the molecular mechanism of FD in alfalfa.

**Table:** 

Specifications	
Organism/cell line/tissue	*Medicago sativa* L. standard varieties Maverick and CUF101
Sex	N/A
Sequencer or array type	Illumina Hiseq 2000
Data format	Raw data: FASTQ files
Experimental factors	Fall dormant vs. nondormant
Experimental features	RNA-seq dataset for gene expression analysis in fall dormant and fall nondormant alfalfa leaves
Consent	N/A
Sample source location	The Experimental Station of Henan Agricultural University, Zhengzhou, China (34°19 × N, 113°35 × E)

## Direct link to deposited data

Deposited data can be found here: https://trace.ddbj.nig.ac.jp/DRASearch/submission?acc=SRA057663.

## Experimental design, materials and methods

### Experimental design

Dormant alfalfa shows fall dormancy (FD) morphology in late summer or early autumn. Shortening day length and falling temperature have been recognized as the environmental factors that induce dormancy in alfalfa [1]. However, the mechanism involved in FD is still not clear. Since the genome of alfalfa has not been sequenced yet, exploring the expression profile of dormant and nondormant alfalfa using RNA-seq may be important for understanding the mechanism of FD in alfalfa.

### Plant materials and total RNA isolation

Alfalfa standard varieties Maverick and CUF101 were planted at the Experimental Station of Henan Agricultural University, Zhengzhou, China. D5, ND5, D9, and ND9 were used as abbreviations for dormant type (Maverick) in May, nondormant type (CUF101) in May, dormant type in September, and nondormant type in September, respectively (Table 1). Detailed growth conditions in our study were described by Zhang et al. (Zhang S et al., De novo Characterization of Fall Dormant and Nondormant Alfalfa (*Medicago sativa* L.) Transcriptome and Identification of Candidate Genes Relate to Fall Dormancy, submitted for publication).

Alfalfa leaves were collected both on May 19 and September 23, 2011, fourteen days after alfalfa was harvested. Leaves were frozen in liquid nitrogen and then stored at − 80 °C until for total RNA isolation. After grinding tissues in pre-chilled (using liquid nitrogen) pestle and mortar, total RNA was isolated from each sample using TRIzol Reagent (Invitrogen) according to the manufacturer's instruction. RNase-free DNase I (Fermentas) was used to remove contaminant genomic DNA in the isolated total RNA. Nanodrop 2000 (Thermo) was used to measure total RNA concentration and the OD260/280 value was accepted between 1.8 and 2.1. RNA integrity number (RIN) was identified using Agilent 2100 Bioanalyzer (Agilent) and each sample had a RIN value of > 7.5.

### Generation of sequencing data

To obtain the whole expression profiles at mRNA level, RNA-seq was conducted in eight sequencing libraries (four samples, each sample had two biological replicates). Libraries were prepared according to TruSeq RNA Sample Prep v2 LS Protocol (Illumina). Paired-end sequencing of 100 bp was performed using Hiseq 2000 (Illumina). After Illumina's standard filtering high quality sequence reads were obtained in FASTQ format and submitted to NCBI Sequence Read Archive (SRA) (accession number SRA057663).

### Gene expression analyses

Primers/adaptors were removed from raw sequencing reads. Quality of sequencing reads was examined using FastQC tool [2]. An average Phred quality score of 38 was achieved per sequencing read, indicating high quality sequence reads. Since the genome of alfalfa is not available at present, we put all of sequencing reads generated in our study (160 million in total) together to assemble a reference transcriptome using Trinity (version r2012-05-18) [3]. Sequencing reads generated from each sample was aligned to the reference transcriptome using Bowtie (version 0.12.8) [4] individually, this was done using a Perl script (alignReads.pl) involved in Trinity with default parameters. In each of the eight sequencing samples, about 70% of reads were mapped back on the reference transcriptome successfully. Read counts were estimated using a modified RSEM (version 1.1.18) [5] in Trinity.

Differential gene expression analysis between the four conditions (D5 vs. ND5, D9 vs. ND9, D5 vs. D9 and ND5 vs. ND9) were conducted using Simbiot® [6] platform. Gene expression analysis was based on raw count data generated by RSEM mentioned above. The count data was formatted into a CSV file and uploaded to the Simbiot® platform, all further processing was performed within the platform. The count data were constructed into data matrixes and transformed using DESeq [7] variance reduction algorithm. The transformed matrixes were normalized using quantile algorithm built into the Bioconductor [8] Limma package [9] with default option. Expression analysis was performed using Limma with Benjamini and Hochberg fall discovery control procedure. Gene expression results with fold change ≥ 2 and adjusted *P* value ≤ 0.01 were considered as differentially expressed using R [10]. As a result, 1841 DE genes were at the May time point between dormant (D5) and non-dormant (ND5) lines. Likewise, 2064 genes were different between the genotypes at the September time point (D9 vs. ND9). A comparison between the two time points in the same cultivars identified 1780 and 1652 genes for the D5 vs. D9 and ND5 vs. ND9 cultivars respectively. Besides, gene ontology (GO) [11] enrichment analyses were studied using BiNGO (version 3.0.2) [12] plugin in Cytoscape (version 3) [13], with the Benjamini and Hochberg False Discovery Rate (FDR) correction and the significant level of 0.05. Kyoto Encyclopedia of Genes and Genomes (KEGG) Automatic Annotation Server (version 1.67x) to retrieve the KEGG orthology (KO) assignments and KEGG pathways, using single-directional best hit (SBH) assignment method.

**Fig. 1:**
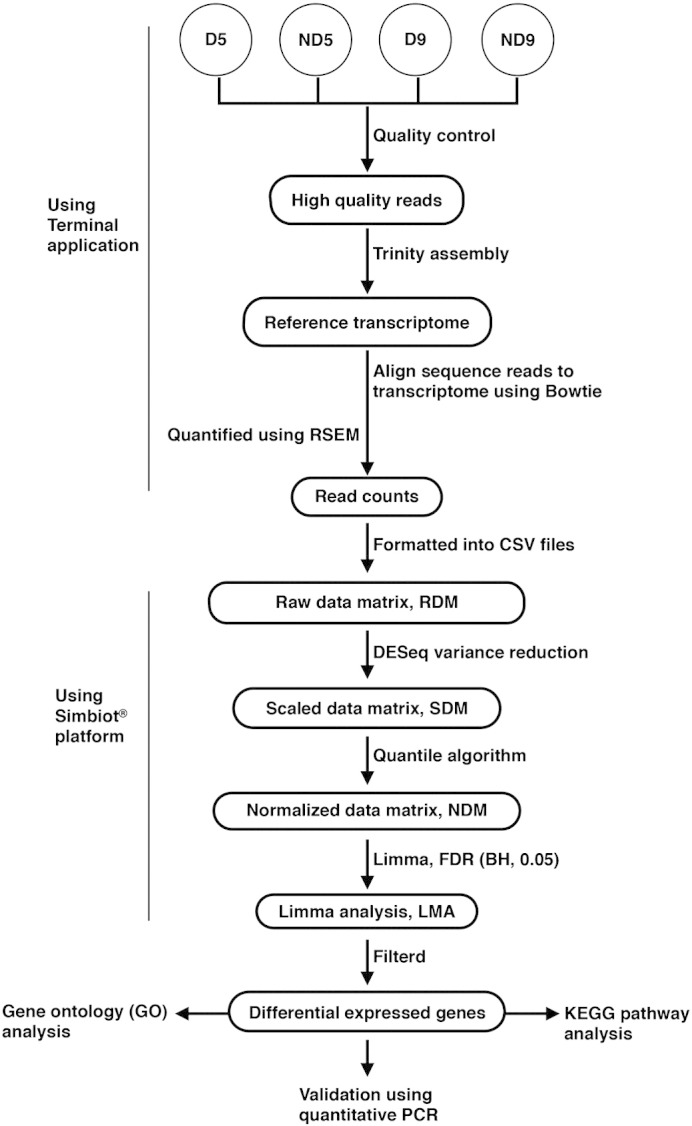
Flow chart of gene expression analysis in the study. CSV, comma-separated values.

Quantitative PCR was performed to validate the DE genes generated in our study. Twenty-two differential genes were selected and the results showed a high correlation (R^2^ = 0.73) of fold change between sequencing and quantitative PCR. The flow chart of gene expression analysis was depicted in Fig. 1.

## Discussion

We described here a unique RNA-seq dataset of alfalfa. This dataset is composed of fall dormant and nondormant alfalfa varieties at two time points. It revealed differential gene expression patterns involved in fall dormancy of alfalfa. This genomic resource, combined with previous studies on fall dormancy research in alfalfa, will help us get a closer look at fall dormancy regulation network in alfalfa.

## Conflict of interest

The authors declare no conflict of interest.

## Figures and Tables

**Table 1:** Samples used for RNA-seq.

Variety	May	September
Maverick (FDC1)	D5	D9
CUF101 (FDC9)	ND5	ND9

Abbreviations: FDC, fall dormancy class, ranging from 1 to 11, to determine whether a variety is fall dormant type or not.

In this study, FDC1 represents fall dormant type and FDC9 represents fall nondormant type.
